# Stabilization of soluble high‐affinity T‐cell receptor with *de novo* disulfide bonds

**DOI:** 10.1002/1873-3468.13616

**Published:** 2019-10-08

**Authors:** Flávio Sádio, Gerhard Stadlmayr, Katharina Stadlbauer, Maximilian Gräf, Agnes Scharrer, Florian Rüker, Gordana Wozniak‐Knopp

**Affiliations:** ^1^ Christian Doppler Laboratory for Innovative Immunotherapeutics Department of Biotechnology University of Natural Resources and Life Sciences (BOKU) Vienna Austria

**Keywords:** novel disulfide bond, pMHC binding, soluble TCRs, stability engineering

## Abstract

Soluble T‐cell receptors (TCRs) have recently gained visibility as target‐recognition units of anticancer immunotherapeutic agents. Here, we improved the thermal stability of the well‐expressed high‐affinity A6 TCR by introducing pairs of cysteines in the invariable parts of the α‐ and β‐chain. A mutant with a novel intradomain disulfide bond in each chain also tested superior to the wild‐type in the accelerated stability assay. Binding of the mutant to the soluble cognate peptide (cp)–MHC and to the peptide‐loaded T2 cell line was equal to the wild‐type A6 TCR. The same stabilization motif worked efficiently in TCRs with different specificities, such as DMF5 and 1G4. Altogether, the biophysical properties of the soluble TCR molecule could be improved, without affecting its expression level and antigen‐binding specificity.

## Abbreviations

**DSC**, differential scanning chromatography

**ER**, endoplasmic reticulum

**Fc**, fragment crystallizable

**NTA**, nitrilotriacetic acid

**pMHC**, peptide–major histocompatibility complex

**PNGase F**, peptide:N‐glycosidase F

**RT**, room temperature

**SEC**, size exclusion chromatography

**TCR**, T‐cell receptor

***T*_m_**, melting temperature

**TNB**, 2‐nitro‐5‐thiobenzoic acid

T‐cell receptors (TCR) have evolved to recognize peptide–MHC molecules on the cell membranes. Their use for therapeutic purposes has traditionally relied on their expression as membrane‐bound molecules expressed on the surface of T lymphocytes with TCR α‐ and β‐variable domains used for antigen recognition 1, 2. Due to their unique target repertoire not accessible to antibodies, soluble TCRs are attractive targeting moieties of agents that have already entered clinical trials 3. Before reaching this stage, important modifications of the molecule were required, primarily directed toward improving their stability 4. The polypeptide linkage between the variable α‐and β‐ domains allowed their display as single‐chain TCRs on the surface of phage particle 5 or yeast cell 6, and directed evolution of the derived libraries enabled the increase of their intrinsic low micromolar binding affinity toward the cognate antigen for several orders of magnitude 7, 8. These improved properties promise to expand their potential use in adoptive cell therapies 9, 10, but also enabled their clinical application as soluble fusion proteins with a single‐chain antibody specific for CD3ε subunit 11, 12, 13. TCRs act as bridging molecules between a tumor cell expressing cp–major histocompatibility complex (pMHC) and effector T cells, elicit T‐cell activation upon encountering tumor cell, and thus cause potent target cell killing. This approach proved invaluable in targeting tumor cells that cannot be recognized by an antibody as the relevant peptide sequence originates from an intracellular antigen, such as in the case of melanoma and prostate cancer 14.

The TCR elements concerned with antigen binding include primarily the α‐ and β‐ variable domains, which has triggered the development of soluble TCR formats, such as α/β heterodimer or only the variable domains connected with a polypeptide linker sequence 15, 16, 17, a disulfide bond 4, 18 or another dimerization agent, such as the constant domain of antibody kappa‐light chain 19. Constant domains of α‐ and β‐chain are naturally connected with a C‐terminal disulfide bond, analogous to the one present in the Fab fragment of IgG1 antibodies. Folding of the TCR in the endoplasmic reticulum (ER) is critically dependent on the formation of the α/β heterodimer 20, especially the folding of the TCR constant domain Cα, which displays little homology to other Ig domains and strongly deviates from the Ig fold 21.

Although TCRs share the basic immunoglobulin fold with the antibodies, their biophysical properties make their manufacturing difficult: Typically, the expression yields are low and purified product exhibits misfolding and aggregation. This was described for both *Escherichia coli* expression systems, which rely on the expression of cytoplasmic inclusion bodies 22 or periplasmic TCR production 23, and mammalian cells. Several experimental setups aimed at improving their thermostability, which has also often positively influenced their solubility and expression level 24, 25. Early efforts to improve the stability of the molecule used obliteration of the C‐terminal residues of α‐ and β‐chain including the interdomain disulfide bond 26. Others applied rational mutagenesis oriented toward reducing hydrophobicity of amino acid residues in variable and in constant domains 24, 27. One interesting approach involved using yeast metabolism to select for better folded TCR molecules that can pass the ER quality control of the yeast cell and resist protease degradation, and were displayed on the cell surface as functional antigen‐binding molecules when yeast cultures are induced at a stress temperature of 37 °C 28. Alternatively, the selection was governed to identify yeast surface display library members that react with a clonotypic anti‐TCR antibody or pMHC antigen after induced yeast cells have been incubated at a high temperature after induction 27, 28, 29. Another approach involved producing fusions with the immunoglobulin G fragment crystallizable (Fc) to increase the TCR expression level and solubility, but introduced bivalent pMHC binding 24, 30. Nevertheless, facilitating the production of a soluble TCR that can be compared with the yield of an antibody molecule is still an engineering challenge.

We have chosen an approach where we have screened the constant domains of a soluble α/β‐TCR molecule for the ability to accommodate a novel disulfide bond, using pairwise substitutions of amino acids for cysteine residues. The well‐soluble, well‐expressed, affinity‐optimized anti‐human T‐lymphotropic virus TCR A6 31 with constant domains already connected with a non‐native disulfide bond 4 was used as a starting point for further stabilization. We tested both intradomain and interdomain bonds proposed *in silico*, but restricted the screen to the mutants where the novel disulfide bond either connects variable and constant domains or is positioned within the constant domains of the TCR in order to minimize the effect on antigen binding. We have characterized the protein harboring the identified beneficial stabilization motif for the expression level, correctness of heterodimerization, monomer content, and antigen binding. The effect of cysteine substitutions was also examined in two different soluble TCR scaffolds. Finally, we explored how the effect of the increased melting temperature (*T*_m_) influences the TCR stability in PBS at high temperature.

## Materials and methods

### Design and construction of mutants

The sequences of A6, DMF5, and 1G4 TCRs were as published by Cole *et al.* 31, Borbulevych *et al.* 32 and Dunn *et al.* 33 (Table S1). The gene fragments were purchased from GeneArt. Crystal structures of the A6 (4GRM), DMF5 (3QDG), and 1G4 (2F54) TCRs were visualized with PyMOL (Schrödinger LLC., New York, NY, USA) The algorithm MODIP of DSDBASE (http://caps.ncbs.res.in/dsdbase/dsdbase.html) was used to analyze A6 TCR crystal structure (PDB: 4GRM) to predict the positions that could potentially be mutated to cysteine residues suitable for the creation of intradomain or interdomain disulfide bonds (Table S2). Mutagenesis was performed using QuikChange Lightning Mutagenesis Kit (Agilent, Santa Clara, CA, USA), exactly according to manufacturer’s instructions with oligonucleotides listed in Table S3.

### Expression and purification

pTT‐based expression vectors (CNRC) pTT5 and pTT22SSP4, which encodes an N terminally appended his‐tag, were used for cloning of α‐ and β‐chain of the TCR A6. For expression screening, proteins were produced using PEI‐mediated transient transfection of HEK293‐6E cells (CNRC) according to the manufacturer’s instructions. Two days post‐transfection, cells were fed trypton TN‐20 to a final concentration of 2.5% and supernatant was harvested 5 days post‐transfection with centrifugation at 1300 ***g***, 15 min at 4 °C, and immediately processed or used for further analysis.

Expressible TCR mutants were produced in ExpiCHO system (Thermo Fisher Scientific, Waltham, MA, USA) following the MaxTitre protocol. Supernatants were harvested 12–14 days post‐transfection with centrifugation at 1300 ***g***, 20 min at 4 °C, and further clarified with a centrifugation step at 14 400 ***g***, 20 min at 4 °C. After buffering to PBS, pH 7.5, the supernatant was passed over Excel Ni‐ nitrilotriacetic acid column (GE Healthcare, Chicago, IL, USA) equilibrated with the same buffer. The column was then washed with 10 column volumes of PBS/20 mm imidazole, pH 7.5, and his‐tagged A6 variants were eluted with a gradient from 20 to 500 mm imidazole in five column volumes. Fractions containing the target protein were pooled and dialyzed against a 100‐fold volume of PBS overnight at 4 °C. The proteins were stored at −80 °C until use.

### Biophysical properties

#### SDS/PAGE

Clarified cell culture supernatants or purified protein preparations were mixed with Sample Loading Dye (Thermo Fisher) and analyzed *via* PAGE. Proteins along with the Mark 12 Unstained Standard were resolved on 4–12% Novex NuPAGE® gels, run in morpholino ethanesulfonic acid buffer at 200 V for 35 min, stained with Colloidal Blue Staining Kit (Thermo Fisher Scientific), and destained with distilled water.

#### SEC‐HPLC

Shimadzu LC‐20A Prominence system equipped with a diode array detector and a refractive index detector was used to perform size exclusion chromatography (SEC)‐HPLC with a Superdex 200 Increase 10/300 GL column (GE Healthcare). The mobile‐phase buffer used was PBS with 200 mm NaCl. Chromatography was conducted with a constant flow rate of 0.75 mL·min^−1^. A total of 20 µg protein at about 1 mg·mL^−1^ were loaded on the column for analysis. Column calibration was performed with a set of molecular weight standards ranging from 10 to 500 kDa (Bio‐Rad, Hercules, CA, USA).

#### DSC

Differential scanning chromatography (DSC) experiments were performed using an automated MicroCal PEAQ‐DSC Automated system (Malvern), using 5 µm protein solution, diluted in PBS at pH 7.5. The heating was performed from 20 to 100 °C at a rate of 1 °C·min^−1^. Protein solution was then cooled *in situ*, and an identical thermal scan was run to obtain the baseline for subtraction from the first scan. All measurements were taken at least in triplicates. Fitting was performed with MicroCal PEAQ‐DSC software using the non‐2‐state transition mechanism.

#### Circular dichroism

The far UV‐CD spectra were recorded on a Chirascan spectropolarimeter (Applied Photophysics, Leatherhead, UK) at 25 °C using 1‐mm‐quartz cuvette, with 1 nm step and 0.5 nm slit. Protein preparations were measured in 10 mm sodium phosphate buffer, pH 7.4, at 200 µg·mL^−1^. The CD spectra of the buffer solution were subtracted from the sample spectra before conversion to CD absolute units.

#### Mass spectrometry

Twenty microliter PNGase F digested sample (β = 0.30 mg·mL^−1^) was analyzed using a Dionex Ultimate 3000 system directly linked to a QTOF instrument (maXis 4G ETD; Bruker, Billerica, MA, USA) equipped with the standard ESI source in the positive ion mode. MS scans were recorded within a range from 400 to 3800 *m/z*. Instrument calibration was performed using ESI calibration mixture (Agilent). For separation of the proteins, a Thermo ProSwift™ RP‐4H Analytical separation column (250 × 0.200 mm) was used. A gradient from 80% solvent A and 20% solvent B (A: 0.05% trifluoroacetic acid, B: 80% acetonitrile, and 20% A) to 65% B in 20 min was applied, followed by a 15‐min gradient from 65% B to 95% B, at a flow rate of 8 μL·min^−1^ and at 60 °C. Deconvolution of summed spectra was done using the MaxEnt algorithm in Data Analysis 4.0.

#### Ellman’s assay

To determine the concentration of free sulfhydryl groups in the protein preparations, 0.25 mL of tested protein solution was combined with 0.5 mL reaction buffer (0.1 mm sodium phosphate buffer/1 mm EDTA, pH 8.0) and 50 µL Ellman’s reagent solution [4 mg 2‐nitro‐5‐thiobenzoic acid (TNB; Thermo Fisher Scientific), dissolved in 1 mL reaction buffer] and incubated at RT for 15 min. Absorbance at 412 nm was determined with NanoDrop spectrophotometer. Standard curve was obtained using DTT in 2.5‐fold serial dilutions, ranging from 0.8 to 500 µm. Concentration of free sulfhydryl groups in the sample was determined using the molar extinction coefficient of TNB (14 150 m ^−1^·cm^−1^).

### Binding properties

#### pMHC binding

Peptide–major histocompatibility complex binding of the A6 and DMF5 TCR was evaluated in an ELISA experiment. One hundred microliters of biotinylated pMHC (Immunitrack A/S, Copenhagen, Denmark), diluted in PBS to 5 µg·mL^−1^, was used to coat the wells of the streptavidin‐activated Immobilizer Maxisorp plate (NUNC, Roskilde, Denmark) for 1 h at RT. Plates were then blocked with 200 µL of 4% BSA‐PBS for 1 h, washed three times with 200 µL PBS, and purified preparations of TCR or clarified supernatants containing TCR in 2% BSA‐PBS were allowed to bind for 90 min at RT. After three washes with 200 µL PBS, 100 µL of anti‐penta‐his–horseradish peroxidase conjugate (QIAgen, Hilden, Germany) was applied at 1 : 3000 dilution in 2% BSA‐PBS for 45 min. Binding was revealed with 100 µL 3,3',5,5'‐tetramethylbenzidine (Sigma‐Aldrich, St. Louis, MO, USA), the reaction was stopped using 100 µL of 30% H_2_SO_4_, and the absorbance was determined at 450/620 nm. The dose–response curve was fit with the prism 5 (Graphpad Software Inc., La Jolla, CA, USA) algorithm as one‐site binding with Hill slope. TCR concentration was determined from the ELISA signal of the particular preparation.

The kinetic parameters of pMHC binding were determined using Octet RED96 system (Pall ForteBio, Fremont, CA, USA). Streptavidin tips, equilibrated in assay buffer (PBS with kinetic buffer) (Pall ForteBio), were loaded with 5 µg·mL^−1^ biotinylated pMHC (Immunitrack A/S) for 300 s with agitation at 17 ***g***. After the recoding of second baseline, A6‐ and DMF5‐based TCRs in twofold serial dilutions starting from 200 nm were allowed to bind for 900 s and the tips were then immersed into assay buffer for 600 s for dissociation. Sensorgram curves resulting from TCR dilutions binding to noncoated tips and pMHC‐coated tip immersed into PBS in the association and dissociation step were subtracted as background before fitting the response curves with a 1 : 1 binding model.

#### Cell surface binding

T2 cells (174 × CEM.T2) (ATCC® CRL‐1992™) were cultured in Dulbecco’s modified Eagle medium with 20% FBS and penicillin–streptomycin in humidified atmosphere at 37 °C under 5% CO_2_. For peptide loading, protocol used by Al Qudaihi *et al.* 34 was used. Cells were harvested, washed in PBS, and resuspended to a density of 2.5 × 10^5^ cells in 200 µL medium in the wells of a 96‐well plate. To optimize the peptide concentration for loading, cells were incubated with a fivefold serial dilution of the A6‐cp dissolved in DMSO overnight at 37 °C in humidified atmosphere with 5% CO_2_ for 18 h. Treated cells were then blocked in 2% BSA‐PBS for 30 min on ice. Two hundred five thousand cells/well of a 96‐well plate were stained with threefold serial dilution, starting from 100 nm the wild‐type A6 or DMF5 TCR or their stabilized mutants in 2% BSA‐PBS for 30 min on ice, and 300 nm concentration was used for staining with 1G4 TCR. After a brief wash in ice‐cold PBS, a 1 : 1000 dilution of anti‐his‐Alexa Fluor 647 (QIAgen) in 2% BSA‐PBS was used to detect test protein binding. Dead cells were excluded from the measurement by staining with 1 : 200 solution of *7*‐aminoactinomycin D (Becton Dickinson, Franklin Lakes, NJ, USA) in PBS, and mean fluorescent intensity values of the live cell population were recorded. As 40 µm concentration of the pulsed peptide was the highest not detrimental to cell viability, this was the one used for future cell‐binding experiments.

### Accelerated stability assay

To test the stability of TCR variants in PBS, proteins were diluted to 133 µg·mL^−1^. Two hundred microliter aliquots were incubated at −80, 4, 20, 37, and 50 °C for 1, 7, and 14 days. The 50 °C‐incubation samples were overlayed with mineral oil (Sigma‐Aldrich) to prevent evaporation. The TCR samples that were incubated at 50 °C for 14 days were analyzed with HPLC‐SEC, CD spectroscopy, and SDS/PAGE, as well as examined for their antigen‐binding properties.

## Results

**Figure 1:**
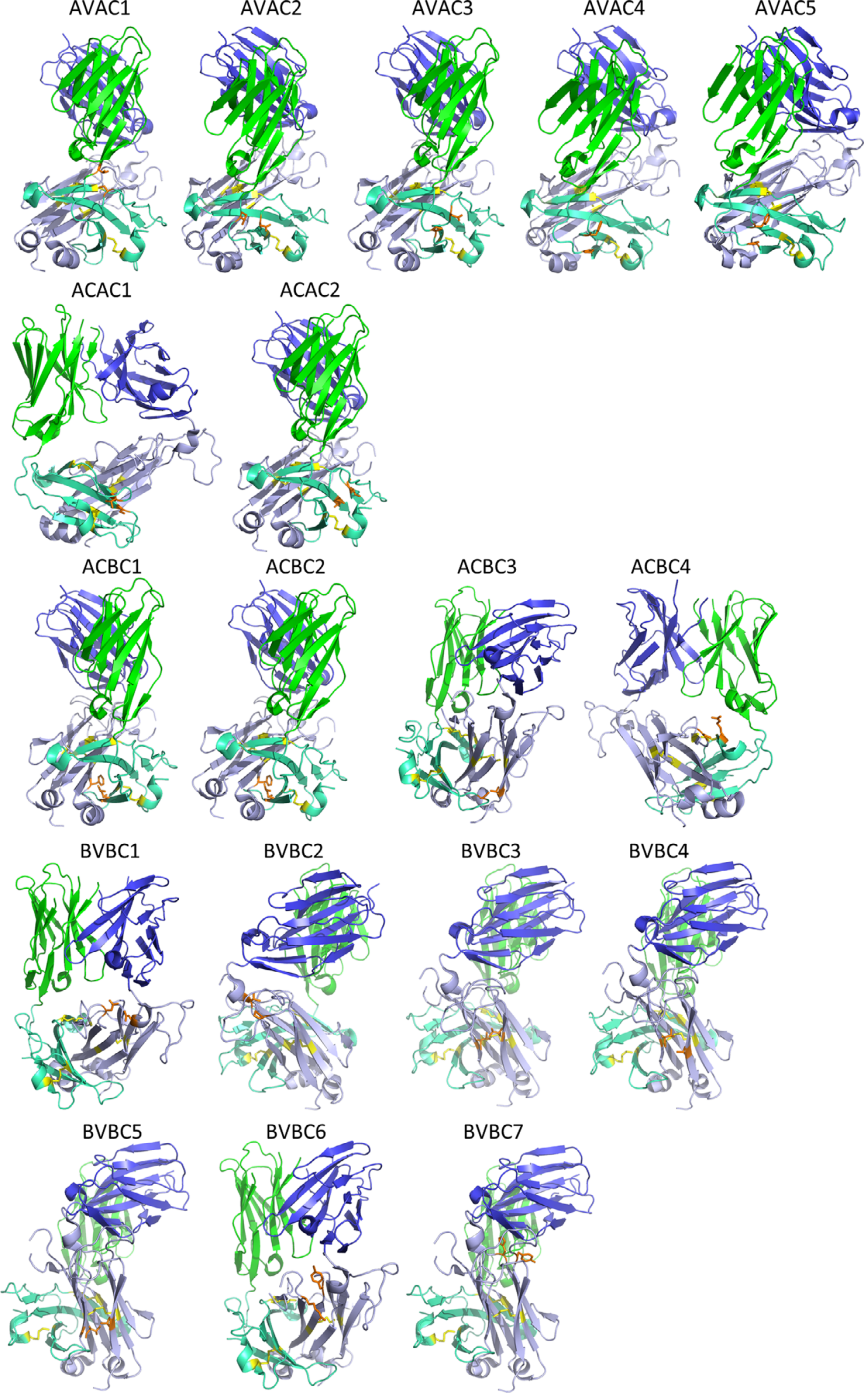
Proposed stabilization mutations in A6 TCR scaffold. Green: variable domain of the α‐chain; teal: constant domain of the α‐chain; blue: variable domain of the β‐chain; light blue: constant domain of the β‐chain; yellow: disulfide bonds present in A6 TCR scaffold; orange: amino acid residues for mutation to cysteine residues. The figure was prepared using PyMOL (Schrödinger LLC.).

In the present work, we aimed to stabilize a TCR molecule by the introduction of *de novo* intradomain or interdomain disulfide bonds. Initial experiments were performed with A6 TCR, and the effects of identified beneficial stabilizing mutations on the stability and ligand binding were characterized in A6, DMF5, and 1G4 scaffolds. The HEK293‐6E suspension cell line was used to produce the soluble A6 TCR for screening. The preliminary experiment where either α‐ or β‐chain of the TCR was his‐tagged has shown a 10‐fold higher expression of functional TCR with his‐tagged β‐chain, independently of the chain ratio when tested with up to fourfold excess of the β‐chain (Fig. S1). This may be a consequence of the critically decreased solubility of the α‐chain caused by the appended his‐tag, as described before for other proteins 35. Next, we performed a screen of 18 mutants with newly introduced pairs of cysteine residues: In four, an interdomain bond between constant domains of α‐ and β‐chain was proposed, seven of the mutants were designed with an intradomain disulfide bond within the α‐chain, and seven within the β‐chain (Fig. 1). Of the intradomain mutants, five in the α‐chain and all seven in the β‐chain were intended to connect variable and constant domain of the respective chain. The remaining two proposed intradomain mutants in the α‐chain were positioned within amino acid residues of C‐ and D‐strands of the constant domain.

**Figure 2:**
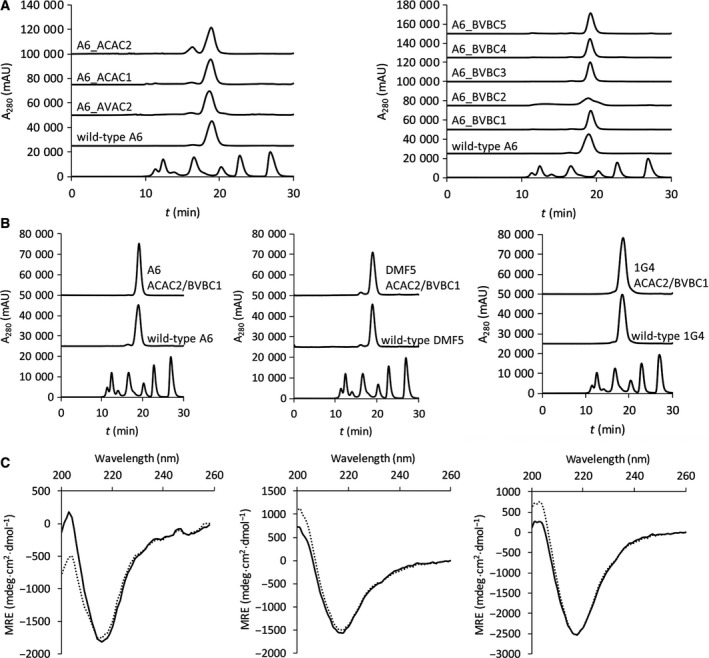
SEC profiles of A6 TCR and its cysteine‐mutated variants modified in A: α‐chain (left panel) or β‐chain (right panel); (B) SEC profiles of A6 (left panel), DMF5 (middle panel), and 1G4 (right panel) TCRs (upper trace: stabilized variants ACAC2/BVBC1; middle trace: wild‐type proteins). Molecular weight marker (Bio‐Rad) (lowest trace) indicates the elution of proteins of 670, 158, 44, 17, and 1.3 kDa; (C) Far UV‐CD spectra of A6 (left), DMF5 (middle), and 1G4 (right) wild‐type (dotted line) and ACAC2/BVBC1 variant (full line).

Supernatants of the transfected HEK293‐6E cells have been analyzed with SDS/PAGE followed by Coomassie staining. Eight of 18 constructed mutants could be expressed at a yield similar as the wild‐type A6 TCR (Table 1 and Fig. S2). Those were then purified using metal affinity chelating chromatography, and 7 displayed wild‐type‐like elution properties in SEC in native conditions (Fig. 2A).

**Table 1:** Putative disulfide bond stabilized mutants of A6 TCR.

Mutant	1st mutated position		2nd mutated position	
	Chain	Amino acid residue	Chain	Amino acid residue
α‐intrachain mutants, connecting variable and constant domain				
AVAC2	α	Ile118	α	Asp145
α‐intrachain mutants within the constant domain				
ACAC1	α	Val158	α	Ser182
ACAC2	α	Ile160	α	Ala180
β‐intrachain mutants				
BVBC1	β	Asn121	β	Asp187
BVBC2	β	Val122	β	Pro232
BVBC3	β	Val127	β	Val237
BVBC4	β	Val127	β	Ala239
BVBC5	β	Val129	β	Ala239

We have next tested the combinations of mutant α‐ and β‐chains for expression, and AVAC2/BVBC1 could be expressed at the wild‐type level, while the level of expression of others did not allow further characterization. The analysis of the immobilized metal affinity chromatography‐purified protein in SEC in native conditions has shown that the preparation was free of aggregates and degradation products as it eluted as a single symmetrical peak, corresponding to the expected size of an assembled dimer (Fig. 2B). Further, mass spectrometry analysis has confirmed the heterodimeric assembly of α‐ and β‐chain identical as in the parental TCR for both molecules (Fig. S3).

Next, we addressed the question whether the identified motif can be tested in a TCR scaffold of another target specificity. The variable domain of the α‐chain of the anti‐Melan MART TCR DMF5 is of the same germline as A6 TCR (TRAV12‐2), but the β‐chain belongs to germline TRBV6‐4/TRBC1 in contrast with TRBV6‐5/TRBC2 of A6 according to IMGT classification 36. The ACAC2/BVBC1 mutant expressed at 11 mg·L^−1^ supernatant, comparable with the wild‐type DMF5 TCR, and had a similar elution profile in HPLC‐SEC in native conditions (Fig. 2B). Similar properties were established also for the mutant of 1G4 TCR (TRAV21‐1/TRBV6‐5/TRBC2) (Fig. 2B). CD spectra of all three mutated variants were similar to the ones of parental TCRs (Fig. 2C). The near‐background values measured for 20–30 µm protein solutions in Ellman’s assay indicated the absence of free sulfhydryl groups and hence pairing of cysteine residues in the tested molecules (for a single free cysteine per molecule an A_412_ value of 0.2–0.5 would be expected).

**Figure 3:**
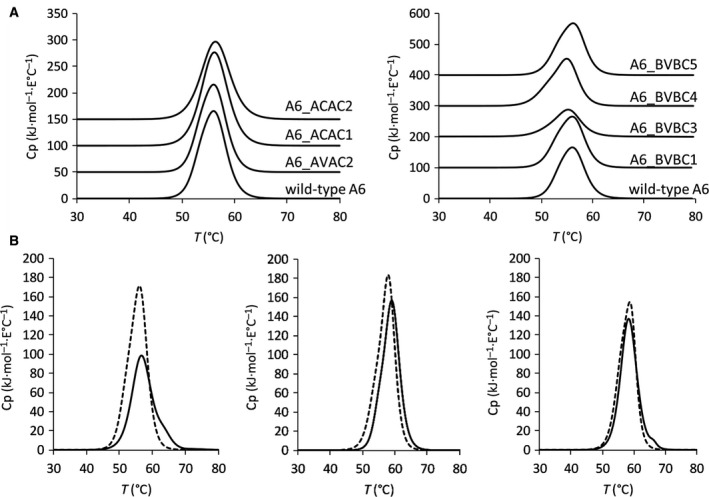
DSC profiles of TCRs and their cysteine‐mutated variants A: A6, modified in α‐chain (left panel), and A6, modified in β‐chain (right panel); (B) A6 (left panel), DMF5 (middle panel), and 1G4 (right panel) wild‐type TCRs (dashed line) and the stabilized variants ACAC2/BVBC1 (full line).

**Figure 4:**
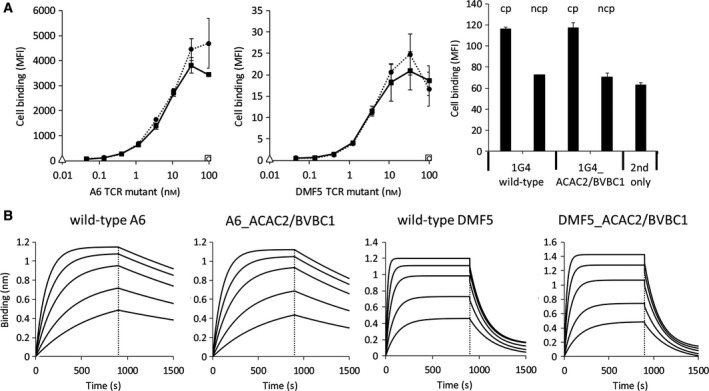
Antigen‐binding properties of A6 and DMF5 TCRs and their stabilized versions; (A) binding to the surface of T2 cells loaded with the cognate peptide of A6 (left panel) and DMF5 (middle panel) wild‐type TCRs and their stabilized versions (dotted line: wild‐type; full line: ACAC2/BVBC1; empty circle: binding of wild‐type TCR to the noncognate peptide (ncp)‐loaded cells; empty square: binding of ACAC2/BVBC1 to the ncp‐loaded cells; empty triangle: cells incubated with secondary reagent only). Antigen reactivity (right panel) of 1G4 was determined at 300 nm TCR binding to T2 cells loaded with cp; ncp; (B) binding to pMHC in ForteBio Octet of A6 and DMF5 TCRs.

Thermostability profile of the wild‐type A6 TCR was determined using DSC: The onset of thermal denaturation was at 42.1 ± 0.19 °C and completed at 62.8 ± 1.8 °C, and the midpoint temperatures of individual melting events of this multidomain protein overlapped to give an endotherm with a single discernible melting point at 55.6 ± 0.1 °C (Fig. 3A). This value was determined to be independent of protein concentration in the range of 3–5 µm with 3 µm being the lowest concentration where signal‐to‐noise ratio was sufficient to obtain reliable data (Fig. S4). The putative novel disulfide‐stabilized candidate mutant was then analyzed with the same protocol and discovered to be of higher thermostability than the wild‐type A6 TCR (Fig. 3B). Thermal denaturation started at 47.58 ± 1 °C and was completed at 70 °C. The DSC profile was deconvoluted with two transitions, one at 56.7 ± 0.08 °C and one at 63.2 ± 0.71 °C. A positive shift of *T*_m_ for 2 °C was established with DSC analysis for the ACAC2/BVBC1 mutant of DMF5, and thermal unfolding of the stabilized mutant of 1G4 was completed at 68.6 ± 0.61 °C in comparison with 66 ± 0.1 °C characteristic for wild‐type protein (Fig. 3B). To show that the mutations have not caused a perturbation of the antigen‐binding site, we have determined the binding to cell‐bound MHC, loaded with exogenous peptide, which was found not to be distinguishable from all three parental TCRs (peptide sequences in Table S1), and the specificity of interaction was confirmed with lack of TCR reactivity with cells, pulsed with an unrelated peptide (Fig. 4A). The kinetic parameters of binding to soluble peptide–MHC for stabilized mutants of A6 and DMF5 TCRs did not deviate from the wild‐type molecule (Fig. 4B and Table 2) (the antigen affinity of 1G4 was insufficient to be reliably measured).

**Table 2:** Kinetic parameters of binding to cognate pMHC of TCRs A6 and DMF5 and their stabilized variants.

	A6			DMF5		
	*K*_D _(nm)	*K*_on _(×10^4^ nm ·s^−1^)	*K*_off _(1/10^4^ s)	*K*_D _(nm)	*K*_on _(×10^4^ nm ·s^−1^)	*K*_off _(1/10^4^ s)
Wild‐type	8.3 ± 0.45	5.08 ± 0.91	4.16 ± 0.48	31.6 ± 7.20	23.6 ± 1.55	0.74 ± 0.12
ACAC2/BVBC1	8.99 ± 2.01	5.87 ± 0.24	5.36 ± 1.44	28.7 ± 0.63	18.90 ± 1.80	0.54 ± 0.039

**Figure 5:**
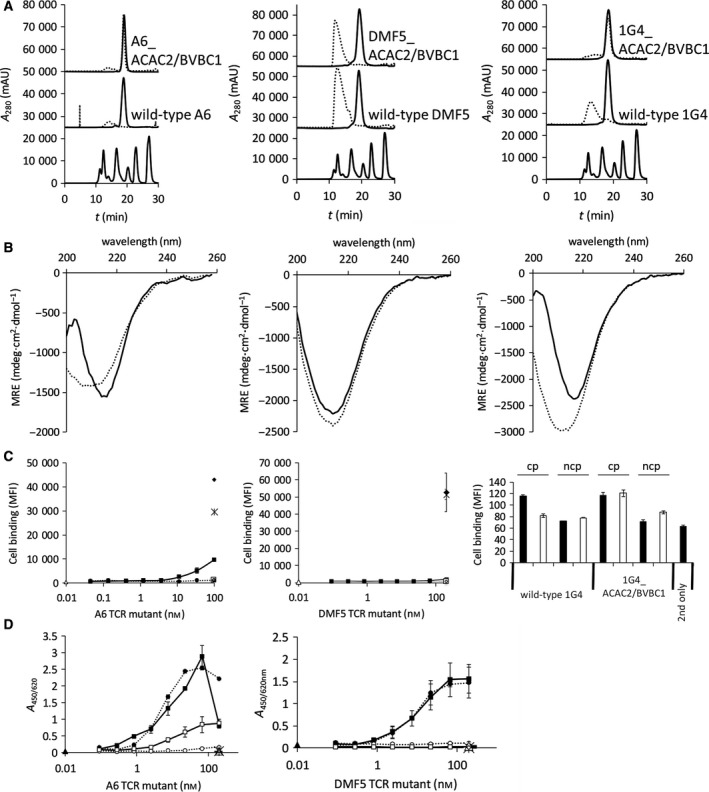
Effect of 14‐day incubation at 50 °C on the biophysical and antigen‐binding properties of wild‐type and stabilized TCR molecules. (A) SEC profiles of A6 (left panel), DMF5 (middle panel), and 1G4 (right panel) TCRs before (dotted line) and after incubation (full line). Molecular weight marker (Bio‐Rad) (lowest trace) indicates the elution of proteins of 670, 158, 44, 17, and 1.3 kDa; (B) Far UV‐CD spectra of treated proteins A6 (left), DMF5 (middle), and 1G4 (right) wild‐type (dotted line) and ACAC2/BVBC1 variant (full line); (C) residual binding to peptide‐loaded cells (dotted line: wild‐type; full line: ACAC2/BVBC1; empty circle: binding of wild‐type TCR to the ncp‐loaded cells; empty square: binding of ACAC2/BVBC1 to the ncp‐loaded cells; asterisk: binding of wild‐type TCR without treatment; full diamond: binding of ACAC2/BVBC1 variant without treatment; empty triangle: cells incubated with secondary reagent only). Right panel shows residual binding of 1G4 TCR at 300 nm before (full bars) and after (empty bars) heat incubation; cp: to cells pulsed with cognate peptide; ncp: to cells pulsed with ncp; (D) residual binding to pMHC in ELISA (dotted line‐full circles: wild‐type without incubation; dotted line‐empty circles: wild‐type with incubation; full line‐full squares: ACAC2/BVBC1 without incubation; full line‐empty squares: ACAC2/BVBC1 with incubation; empty triangle: background binding of wild‐type without incubation; empty diamond: background binding of wild‐type with incubation; asterisk: background binding of ACA2/BVBC1 with incubation; full bar: background binding of ACA2/BVBC1 without incubation; full triangle: secondary reagent only).

We next examined the effect of stabilizing mutations on the TCR in an accelerated stability test. The integrity of protein preparations was monitored after storing them in PBS at temperatures ranging from 4 to 50 °C, over the 14‐day period. While the wild‐type A6 has shown considerable loss of monomeric fraction when incubated at 50 °C for more than 7 days, its thermostabilized variant was still in 94.5% monomeric as determined with HPLC‐SEC in native conditions (Fig. 5A), indicating that its storage stability was improved upon the introduction of the stabilizing motif. Aggregation could also be observed on SDS/PAGE gels (Fig. S5). At lower temperatures, there was no effect on the content of the monomeric fraction for either the wild‐type or the stabilized variant (Fig. S6). Interestingly, the loss of monomeric fraction was similar to the wild‐type in the single disulfide bond‐substituted mutants A6_ACAC2 and A6_BVBC1 (Fig. S6). Beneficial effect of the novel disulfide bonds could be established also for the 1G4 TCR. Similarly as in the A6 scaffold, the loss of monomeric fraction of 1G4_ACAC2/BVBC1 amounted only to 0.6% in contrast to strong aggregation of the wild‐type 1G4 TCR (Fig. 5A). Far UV‐CD spectra of long‐term 50 °C‐incubated wild‐type A6 and 1G4 TCRs have shown a decrease in the negative ellipticity at 218 nm, which indicates a lower content of the β‐sheet‐dominated secondary structure, and an increase in the random coil content in comparison with nontreated proteins. In contrast, the ACAC2/BVBC1 variants of A6 and 1G4 minimum of ellipticity at 218 nm were decreased only by 15% and 7%, respectively (Fig. 5B). While the stabilized A6 variant could still show residual binding to the T2 cell line pulsed with the cp, the wild‐type variant was completely inactivated (Fig. 5C), and similar result was obtained for binding of soluble pMHC tetramers (Fig. 5D). Cell‐bound antigen recognition was also afforded by the long‐term heat‐treated stabilized 1G4 TCR (Fig. 5C). In spite of the positive shift in thermal stability, a beneficial effect on storage stability properties at used conditions could not be established for the DMF5_ACAC2/BVBC1 mutant, as the loss of monomeric fraction, a decrease in the content of secondary structural elements, and obliteration of antigen recognition properties were similar for the wild‐type and the stabilized DMF5 TCR (Figs 5A–D and Fig S5).

## Discussion

In the attempts to improve the properties of a TCR molecule of VαCα/VβCβ format, it has been shown previously that a non‐native disulfide bond between α‐ and β‐chain not only increases the stability of the soluble protein and enhances dimer formation, the receptors stabilized in this way are also more efficiently expressed on the surface of human leukocytes 37, induce cytokine secretion and specific tumor cell lysis more potently, and retain their enhanced expression in CD4+ lymphocytes. In the present work, we have applied the DSDBASE MODIP algorithm to identify novel disulfide bonds in the constant domains of TCRs to further stabilize the scaffold. Surprisingly, of the 18 mutants proposed by the algorithm, only eight could be expressed, and only one mutant with novel disulfide bonds in both α‐ and β‐chains was stabilized. The success of prediction was lower than achieved with DSDBASE algorithm with other proteins, such as the Fc fragment 38 or the large extracellular loop of CD81, an α‐helical protein of the tetraspanin family 39. One reason might be that the chosen algorithm mainly takes into account the parameters of secondary structure provided, and the regions of TCR where the proposed stabilization mutations cluster can be less prominently assigned to such particular elements.

Both prokaryotic and eukaryotic expression systems have proven to be valid sources of soluble TCRs. For prokaryotic systems, refolding has an efficient alternative in periplasmic expression, where the oxidative environment allows the formation of disulfide bonds 40. Periplasmic TCR expression is enhanced if the bacterial secretory pathway is supported by overexpression of folding chaperone proteins 23, 41, and higher stability of the expressed TCR correlates with the fraction of the active product 23. Mammalian expression systems for soluble TCRs could increase in importance in the future as the protein produced in this way offers a more direct comparison with the TCRs selected from mammalian display systems, which are gaining in popularity 42, 43, 44, due to more stringent protein quality control mechanisms that prevent the display of misfolded TCR mutants. For soluble TCRs, the improvement of thermodynamic properties by TCR variable region mutagenesis and the introduction of a disulfide bridge between the TCR constant domains have in many cases been reported to result in an improved yield of the functional TCR secreted from several eukaryotic systems 25, 45. The identified combination of disulfide bonds has not negatively influenced the yield of the TCRs expressed by mammalian cells.

The primary interaction with the pMHC antigen proceeds *via* the six complementarity determining region loops, and the specific interaction with the pMHC is primarily dependent on the peptide–TCR sampling 46. Alterations in specificity of antigen binding can be triggered with modifications of the TCR binding site: In the case of high‐affinity TCRs, it has been proposed that the affinity of the TCRs can correlate with the affinity for the structurally related peptides 47, and this can obstruct the practical application due to increased off‐target toxicity 48, 49. Even thermostabilizing mutations, positioned in the interface of the variable domains of the heterodimer, could enhance antigen binding in spite of a vast spatial difference of 20 Å 29, 50. Importantly, the identified stabilization motif in the constant domains had no effect on the affinity to the cognate pMHC and the molecule retained the specificity of antigen recognition, as proven with an unrelated MHC‐bound peptide. It would, however, be very interesting to study the activity of the proposed stabilized TCRs when introduced into the context of the cell‐bound T‐cell signaling complex, as it has been reported that the conformation of Cβ can critically affect cell signaling *via* the interaction with the components of the CD3 complex 51, 52. As the stabilization motif did not adversely impact the expression level, heterodimerization properties, or the cognate pMHC antigen binding, the novel scaffold is a promising tool for further derivatization of the soluble TCR format, for example, mutagenesis toward functionalization of TCR constant domains for recognition of another antigen, as achieved for immunoglobulin constant domains 53.

To conclude, the TCR scaffold stabilization motif described here can endow the 4‐domain heterodimeric molecule with a superior storage stability and appears functional in different soluble TCRs. Hence, it presents a further step in the development of this promising group of therapeutically valuable targeting agents with improved thermodynamic properties, which could also allow their broader clinical application as soluble reagents.

## Author contributions

GWK and FR conceived and supervised the study; GWK and FS designed experiments; FS, GS, KS, MG, and AS performed experiments; FS, GS, MG, and GWK analyzed data; FS and GWK wrote the manuscript; and GWK and FR made manuscript revisions.

## Funding

The funding source had no influence on study design; on the collection, analysis, and interpretation of data; and on the writing of the report and on the decision to submit the article for publication.

## Supporting information


**Fig. S1.** Expression yields of A6 TCR variants in HEK293‐6E system with his‐tag appended on either ‐ or ‐chain after transfection with different ratios of ‐ to ‐chain.
**Fig. S2.** Results of duplicate test expressions of cysteine‐substituted mutants of A6 TCR analysed with SDS/PAGE.
**Fig. S3.** MS analysis of A6 TCR and the stabilized variant ACAC2/BVBC1.
**Fig. S4.** Concentration dependency of melting profile of wild‐type A6 TCR determined with DSC.
**Fig. S5.** Storage stability of wild‐type and the stabilized variant ACAC2/BVBC1 of TCR A6 (left), DMF5 (center) and 1G4 (right) analyzed with SDS/PAGE after the incubation for the indicated number of days (M: Mark12 molecular weight marker).
**Fig. S6.** A: Storage stability of A6 TCR and the stabilized variant A6_ACAC2/BVBC1 analyzed with SDS‐PAGE at 4, 20 and 37 °C (from left to right) after the incubation for the indicated number of days; B: storage stability of A6 TCR and mutants A6_ ACAC2 (left) and A6_BVBC1 (right) at 50 °C after the incubation for the indicated number of days (M: Mark12 molecular weight marker).
**Table S1.** Amino acid sequence of A6DMF5 and 1G4 TCRs and their respective cognate peptide sequences used.
**Table S2.** A6 TCR mutants proposed for mutagenesis with novel pairs of cysteine residues.
**Table S3.** List of oligonucleotides used for construction of cysteine‐substituted mutants of A6 TCR.
