# Supplementary material for: Stabilization of soluble high‐affinity T‐cell receptor with de novo disulfide bonds
Source: FEBS Lett. 2019 Oct 8;594(3):477–90. doi: 10.1002/1873-3468.13616 (PMC7027902; doi:10.1002/1873-3468.13616)
Supplement: Supplementary file 1 — Fig. S1. Expression yields of A6 TCR variants in HEK293‐6E system with his‐tag appended on either ‐ or ‐chain after transfection with different ratios of ‐ to ‐chain. Fig. S2. Results of duplicate test expressions of cysteine‐substituted mutants of A6 TCR analysed with SDS/PAGE. Fig. S3. MS analysis of A6 TCR and the stabilized variant ACAC2/BVBC1. Fig. S4. Concentration dependency of melting profile of wild‐type A6 TCR determined with DSC. Fig. S5. Storage stability of wild‐type and the stabilized variant ACAC2/BVBC1 of TCR A6 (left), DMF5 (center) and 1G4 (right) analyzed with SDS/PAGE after the incubation for the indicated number of days (M: Mark12 molecular weight marker). Fig. S6. A: Storage stability of A6 TCR and the stabilized variant A6_ACAC2/BVBC1 analyzed with SDS‐PAGE at 4, 20 and 37 °C (from left to right) after the incubation for the indicated number of days; B: storage stability of A6 TCR and mutants A6_ ACAC2 (left) and A6_BVBC1 (right) at 50 °C after the incubation for the indicated number of days (M: Mark12 molecular weight marker). Table S1. Amino acid sequence of A6DMF5 and 1G4 TCRs and their respective cognate peptide sequences used. Table S2. A6 TCR mutants proposed for mutagenesis with novel pairs of cysteine residues. Table S3. List of oligonucleotides used for construction of cysteine‐substituted mutants of A6 TCR. [file FEB2-594-477-s001.pdf]

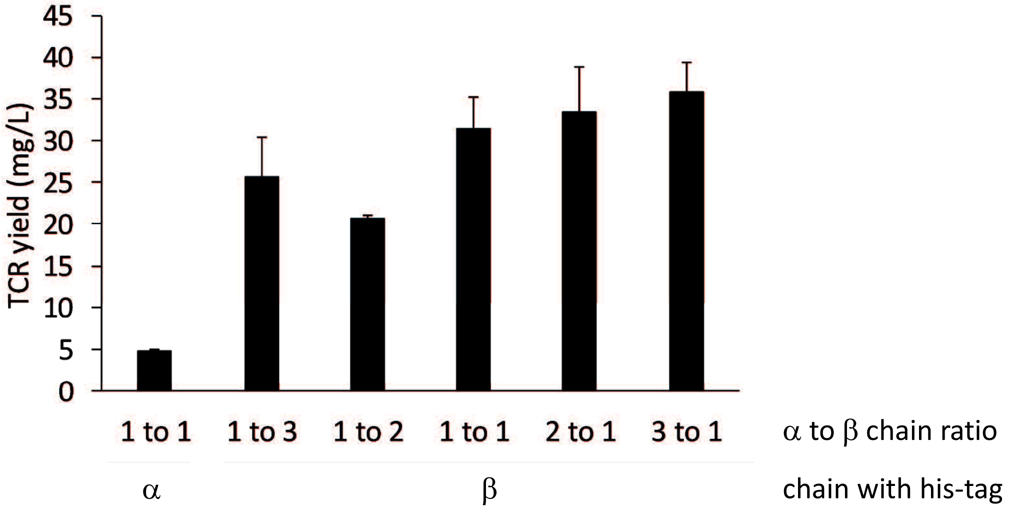

Supplementary Figure 1. Expression yields of A6 TCR variants in HEK293-6E system with his-tag appended on either α- or β-chain after transfection with different ratios of α- to β-chain.

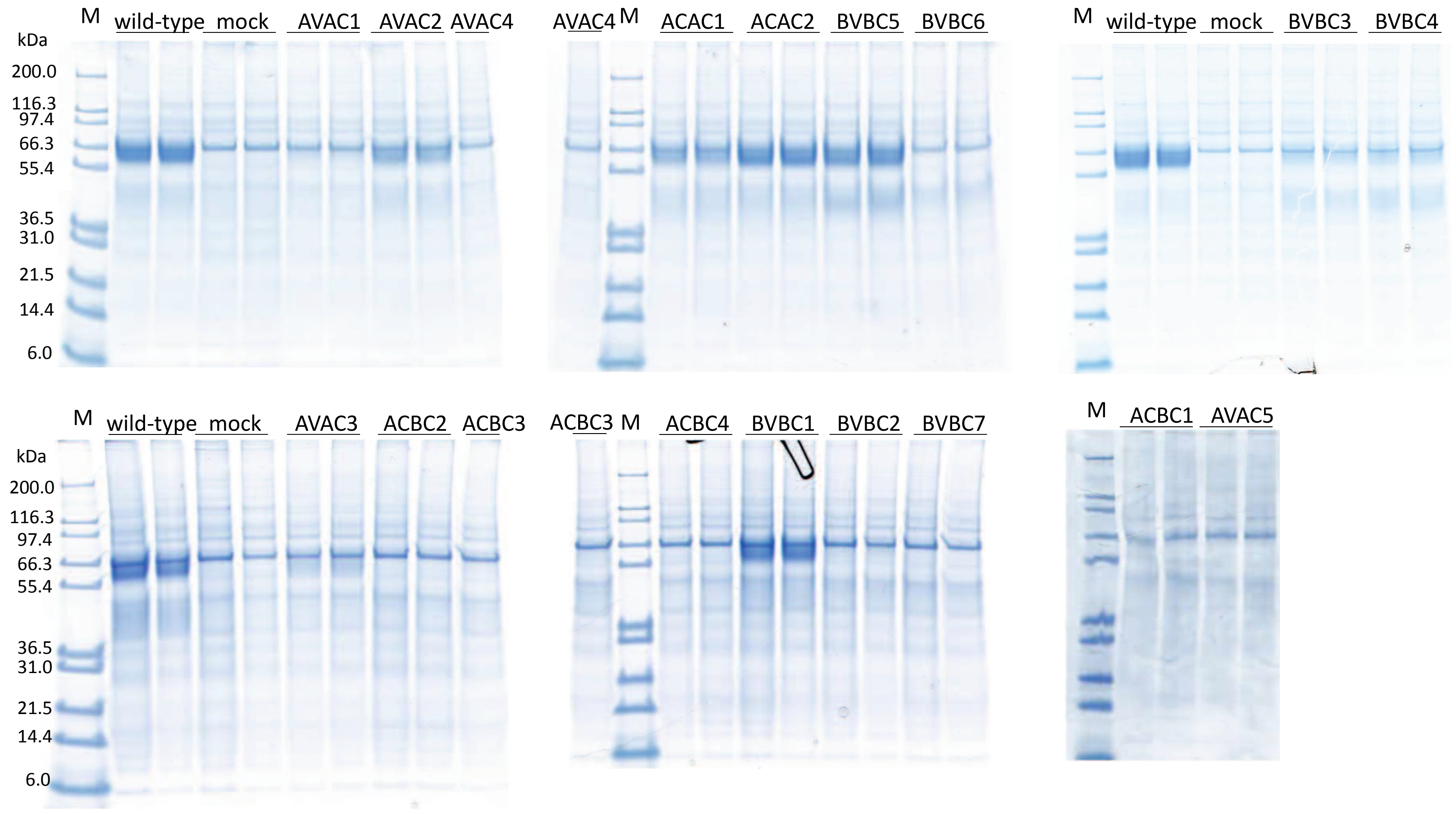

Supplementary Figure 2. Results of duplicate test expressions of cysteine-substituted mutants of A6 TCR analysed with SDS-PAGE.

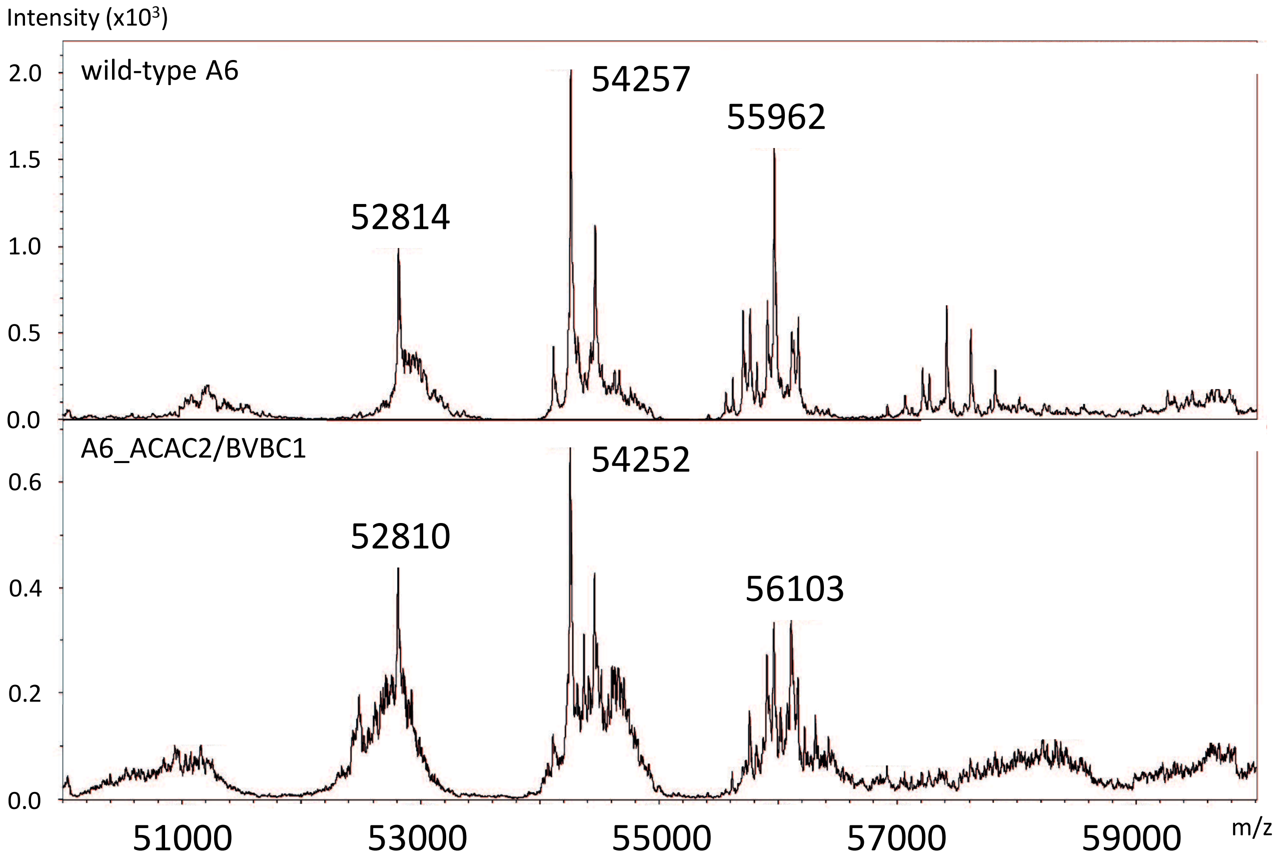

Supplementary Figure 3. MS analysis of A6 TCR and the stabilized variant ACAC2/BVBC1.

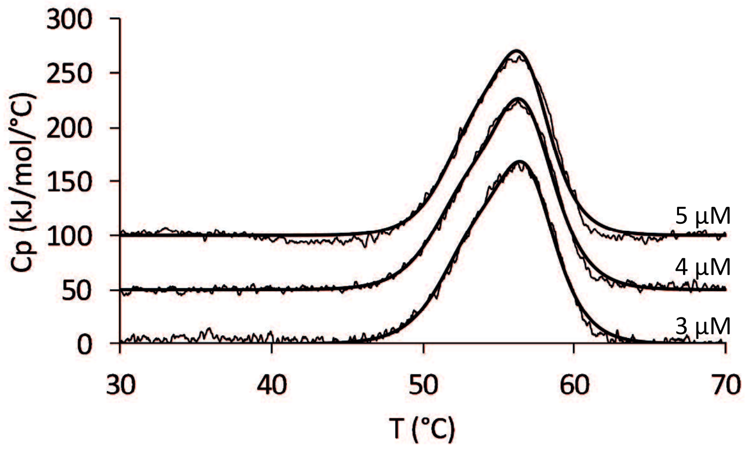

Supplementary Figure 4. Concentration dependency of melting profile of wild-type A6 TCR determined with DSC.

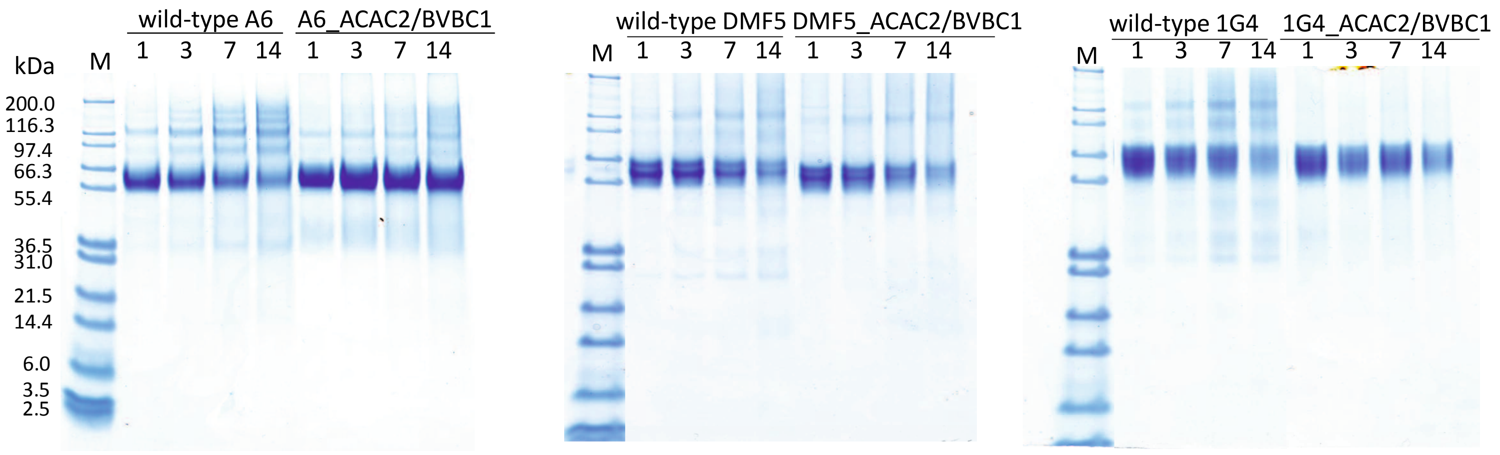

Supplementary Figure 5. Storage stability of wild-type and the stabilized variant ACAC2/BVBC1 of TCR A6 (left), DMF5 (center) and 1G4 (right) analyzed with SDS-PAGE after the incubation for the indicated number of days (M: Mark12 molecular weight marker).

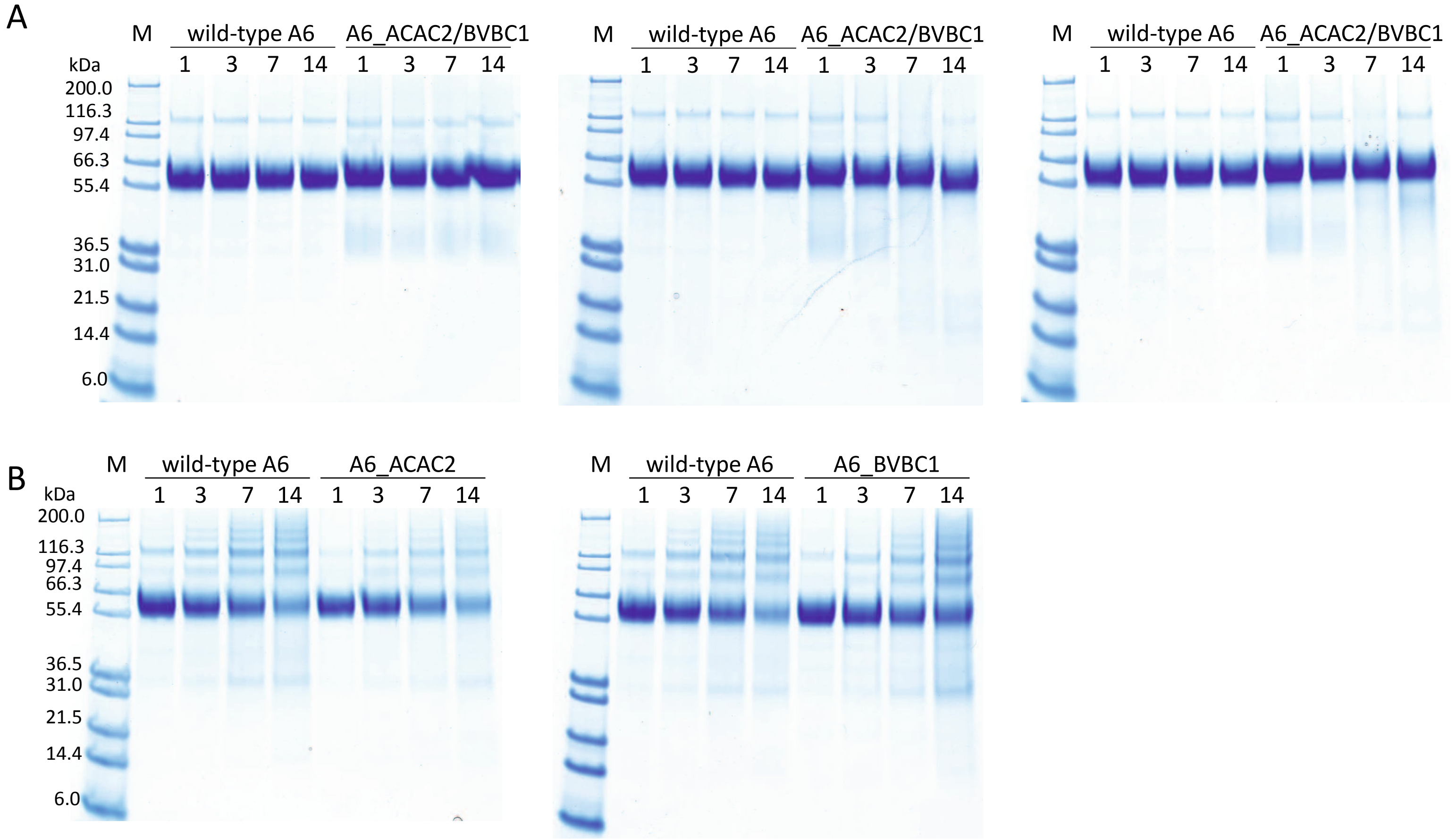

Supplementary Figure 6. A: Storage stability of A6 TCR and the stabilized variant A6_ACAC2/BVBC1 analyzed with SDS-PAGE at 4, 20 and 37 °C (from left to right) after the incubation for the indicated number of days; B: storage stability of A6 TCR and mutants A6_ ACAC2 (left) and A6_BVBC1 (right) at 50 °C after the incubation for the indicated number of days (M: Mark12 molecular weight marker).

A6 TCR		
	α-chain	QKEVEQNSGPLSVPEGAIASLNCTYSDRGSQSFFWYRQYSGKSPELIMSIYSNGDKEDGR<br>FTAQLNKASQYVSLLIRDSQPSDSATYLCAVTTDSWGKLQFGAGTQVVVTPDIQNPDPAV<br>YQLRDSKSSDKSVCLFTDFDSQTNVSQSKDSDVYITDKCVLDMRSMDFKSNSAVAWSNKS<br>DFACANAFNNSIIPEDTFFPS
	β-chain	NAGVTQTPKFQVLKTGQSMTLQCAQDMNHEYMSWYRQDPGMGLRLIHYSVGAGITDQGEV<br>PNGYNVSRSTTEDFPLRLLSAAPSQTSVYFCASRPGLMSAQPEQYFGPGTRLTVTEDLKN<br>VFPPEVAVFEPSEAEISHTQKATLVCLATGFYPDHVELSWWVNGKEVHSGVCTDPQPLKE<br>QPALNDSRYALSSRLRVSATFWQDPRNHFRCQVQFYGLSENDEWTQDRAKPVTQIVSAEA<br>WGRAD
	peptide	LLFGYPVYV
DMF5 TCR		
	α-chain	QKEVEQNSGPLSVPEGAIASLNCTYSYRGSQSFFWYRQYSGKSPELIMFIYSNGDKEDGR<br>FTAQLNKASQYVSLLIRDSQPSDSATYLCAVNFGGGKLIFGQGTELSVKPNIQNPDPAVY<br>QLRDSKSSDKSVCLFTDFDSQTNVSQSKDSDVYITDKCVLDMRSMDFKSNSAVAWSNKSD<br>FACANAFNNSIIPEDTFFPS
	β-chain	IAGITQAPTSQILAAGRRMTLRCTQDMRHNAMYWYRQDLGLGLRLIHYSNTAGTTGKGEV<br>PDGYSVSRANTDDFPLTLASAVPSQTSVYFCASSWSFGTEAFFGQGTRLTVVEDLNKVFP<br>PEVAVFEPSEAEISHTQKATLVCLATGFYPDHVELSWWVNGKEVHSGVCTDPQPLKEQPA<br>LNDSRYALSSRLRVSATFWQDPRNHFRCQVQFYGLSENDEWTQDRAKPVTQIVSAEAWGR<br>AD
	peptide	ELAGIGILTV
1G4 TCR		
	α-chain	KQQVTQIPAALSVPEGENLVLNCSFTDSAIYNLQWFRQDPGGKLTSLLLIQSSQREQTSG<br>RLNASLDKSAGSSTLYIAASQPGDSATYLCAVRPTSGGSYIPTFGRGTSLIVHPYIQNPD<br>PAVYQLRDSKSSDKSVCLFTDFDSQTNVSQSKDSDVYITDKCVLDMRSMDFKSNSAVAWS<br>NKSDFACANAFNNSIIPEDTFFPS
	β-chain	NAGVTQTPKFQVLKTGQSMTLQCAQDMNHEYMSWYRQDPGMGLRLIHYSVGAGITDQGEV<br>PNGYNVSRSTTEDFPLRLLSAAPSQTSVYFCASSYVGNTGELFFGEGSRLTVLEDLKNVF<br>PPEVAVFEPSEAEISHTQKATLVCLATGFYPDHVELSWWVNGKEVHSGVCTDPQPLKEQP<br>ALNDSRYALSSRLRVSATFWQDPRNHFRCQVQFYGLSENDEWTQDRAKPVTQIVSAEAWG<br>RAD
	peptide	SLLMWITQV

Supplementary Table 1. Amino acid sequence of A6DMF5 and 1G4 TCRs and their respective cognate peptide sequences used. The colors correspond to graphical representation of TCR mutants in Figure 1.

Mutant	1st mutated position		2nd mutated position	
	Chain	Amino acid residue	Chain	Aminoacid residue
Proposed α-intrachain mutants, connecting variable and constant domain				
AVAC1	α	Thr115	α	Ser146
AVAC2	α	Ile118	α	Asp145
AVAC3	α	Pro121	α	Asp145
AVAC4	α	Asp122	α	Asp143
AVAC5	α	Pro123	α	Phe144
Proposed α-intrachain mutants within the constant domain				
ACAC1	α	Val158	α	Ser182
ACAC2	α	Ile160	α	Ala180
Proposed α/β-interchain mutants				
ACBC1	α	Tyr126	β	Ser133
ACBC2	α	Leu128	β	Phe130
ACBC3	α	Ser131	β	Ala241
ACBC4	α	Arg169	β	Ser170
Proposed β-intrachain mutants				
BVBC1	β	Asn121	β	Asp187
BVBC2	β	Val122	β	Pro232
BVBC3	β	Val127	β	Val237
BVBC4	β	Val127	β	Ala239
BVBC5	β	Val129	β	Ala239
BVBC6	β	Tyr153	β	Arg189
BVBC7	β	His156	β	Tyr217

Supplementary Table 2. A6 TCR mutants proposed for mutagenesis with novel pairs of cysteine residues.

Supplementary Table 3. List of oligonucleotides used for construction of cysteine-substituted mutants of A6 TCR. For the N121C mutation, a different pair of oligonucleotides was used for 1G4 TCR.

Chain	Mutated position	Oligonucleotides	
α-chain			
	Thr115	A_T115C	ACCCAGGTGGTGGTGTGCCCCGACATCCAGAAC
		A_T115Ca	GTTCTGGATGTCGGGGCACACCACCACCTGGGT
	Ile118	A_I118C	GTGGTGACCCCCGACTGCCAGAACCCCGACCC
		A_I118Ca	GGGTCGGGGTTCTGGCAGTCGGGGGTCACCAC
	Pro121	A_P121C	CCCGACATCCAGAACTGCGACCCCGCCGTGTAC
		A_P121Ca	GTACACGGCGGGGTCGCAGTTCTGGATGTCGGG
	Asp122	A_D122C	GACATCCAGAACCCCTGCCCCGCCGTGTACCAG
		A_D122Ca	CTGGTACACGGCGGGGCAGGGGTTCTGGATGTC
	Pro123	A_P123C	CATCCAGAACCCCGACTGCGCCGTGTACCAGCTG
		A_P123Ca	CAGCTGGTACACGGCGCAGTCGGGGTTCTGGATG
	Tyr126	A_Y126C	CCCGACCCCGCCGTGTGCCAGCTGAGGGACAG
		A_Y126Ca	CTGTCCCTCAGCTGGCACACGGCGGGGTCGGG
	Leu128	A_L128C	CCCGCCGTGTACCAGTGCAGGGACAGCAAGAGC
		A_L128Ca	GCTCTTGCTGTCCCTGCACTGGTACACGGCGGG
	Ser131	A_S131C	GTACCAGCTGAGGGACTGCAAGAGCAGCGACAAG
		A_S131Ca	CTTGTCGCTGCTCTTGCAGTCCCTCAGCTGGTAC
	Asp143	A_D143C	GTGTGCCTGTTCACCTGCTTCGACAGCCAGACC
		A_D143Ca	GGTCTGGCTGTCGAAGCAGGTGAACAGGCACAC
	Phe144	A_F144C	TGCCTGTTCACCGACTGCGACAGCCAGACCAAC
		A_F144Ca	GTTGGTCTGGCTGTCGCAGTCGGTGAACAGGCA
	Asp145	A_D145C	CTGTTCACCGACTTCTGCAGCCAGACCAACGTG
		A_D145Ca	CACGTTGGTCTGGCTGCAGAAGTCGGTGAACAG
	Ser146	A_S146C	GTTCACCGACTTCGACTGCCAGACCAACGTG
		A_S146Ca	CACGTTGGTCTGGCAGTCGAAGTCGGTGAAC
	Val158	A_V158C	GAGCAAGGACAGCGACTGTTATATCACCGACAAG
		A_V158Ca	CTTGTCGGTGATATAACAGTCGCTGTCCTTGCTC
	Ile160	A_I160C	GACAGCGACGTGTATTGTACCGACAAGTGCGTG
		A_I160Ca	CACGCACTTGTCGGTACAATACACGTCGCTGTC
	Arg169	A_R169C	CAAGTGCGTGCTGGACATGTGCAGCATGGACTTCAAG
		A_R169Ca	CTTGAAGTCCATGCTGCACATGTCCAGCACGCACTTG
	Ala180	A_A180C	AACTCCGCGGTGTGCTGGAGCAACAAGAGCGACTTC
		A_A180Ca	GAAGTCGCTCTTGTTGCTCCAGCACACCGCGGAGTT
	Ser182	A_S182C	TCCGCGGTGGCCTGGTGCAACAAGAGCGACTTC
		A_S182Ca	GAAGTCGCTCTTGTTGCACCAGGCCACCGCGGA
β-chain			
	Asn121	B_N121C	GACCGAGGACCTGAAGTGCGTGTTCCCCCCCGAG
		B_N121Ca	CTCGGGGGGGAACACGCACTTCAGGTCCTCGGTC
	Val122	B_V122C	GAGGACCTGAAGAACTGCTTCCCCCCCGAGGTG
		B_V122Ca	CACCTCGGGGGGGAAGCAGTTCTTCAGGTCCTC
	Val127	B_V127C	GTGTTCCCCCCCGAGTGCGCCGTGTTCGAGCCC
		B_V127Ca	GGGCTCGAACACGGCGCACTCGGGGGGGAACAC
	Val129	B_V129C	CCCCCCGAGGTGGCCTGCTTCGAGCCCAGCGAG
		B_V129Ca	CTCGCTGGGCTCGAAGCAGGCCACCTCGGGGGG
	Phe130	B_F130C	CCGAGGTGGCCGTGTGCGAGCCCAGCGAGGC
		B_F130Ca	GCCTCGCTGGGCTCGCACACGGCCACCTCGG
	Ser133	B_S133C	GCCGTGTTCGAGCCCTGCGAGGCCGAGATCAGC
		B_S133Ca	GCTGATCTCGGCCTCGCAGGGCTCGAACACGGC
	Tyr153	B_Y153C	CTGGCCACCGGCTTCTGCCCCGACCACGTGG
		B_Y153Ca	CCACGTGGTCGGGGCAGAAGCCGGTGGCCAG
	His156	B_H156C	GGCTTCTACCCCGACTGCGTGGAGCTGAGCTGG
		B_H156Ca	CCAGCTCAGCTCCACGCAGTCGGGGTAGAAGCC
	Ser170	B_S170C	GGCAAGGAGGTGCACTGCGGCGTGTGTACAG
		B_S170Ca	CTGTACACACGCCGCAGTGCACCTCCTTGCC
	Asp187	B_D187C	CAGCCCGCCCTGAACTGTAGCAGGTACGCCCTG
		B_D187Ca	CAGGGCGTACCTGCTACAGTTCAGGGCGGGCTG
	Arg189	B_R189C	GCCCTGAACGACAGCTGTTACGCCCTGAGCAGCAGGCTG
		B_R189Ca	CAGCCTGCTGCTCAGGGCGTAACAGCTGTCGTTCAGGGC
	Tyr217	B_Y217C	TGCCAGGTGCAGTTCTGCGGCCTGAGCGAGAACGAC
		B_Y217Ca	GTCGTTCTCGCTCAGGCCGCAGAACTGCACCTGGCA
	Pro232	B_P232_C	CAGGACAGGGCCAAGTGCGTGACCCAGATCGTG
		B_P232_Ca	CACGATCTGGGTCACGCACTTGGCCCTGTCCTG
	Val237	B_V237C	CCCGTGACCCAGATCTGCAGCGCCGAGGCCTGG
		B_V239Ca	CCAGGCCTCGGCGCTGCAGATCTGGGTCACGGG
	Ala239	B_A239C	GTGACCCAGATCGTGAGCTGCGAGGCCTGGGGCAG
		B_A239Ca	CTGCCCCAGGCCTCGCAGCTCACGATCTGGGTCAC
	Ala241	B_A241C	GATCGTGAGCGCCGAGTGCTGGGGCAGGGCCGAC
		B_A241Ca	GTCGGCCCTGCCCCAGCACTCGGCGCTCACGATC
	Asn121 in 1G4	B_N121C	GCTGGAGGACCTGAAGTGCGTGTTCCCCCCCGAG
		B_N121Ca	CTCGGGGGGGAACACGCACTTCAGGTCCTCCAGC
