# Supplementary material for: Stabilization of soluble high‐affinity T‐cell receptor with de novo disulfide bonds
Source: FEBS Lett. 2019 Oct 8;594(3):477–90. doi: 10.1002/1873-3468.13616 (PMC7027902; doi:10.1002/1873-3468.13616)
Supplement: Supplementary file 2 [file FEB2-594-477-s002.docx]

Supplementary material

Supplementary Figure 1. Expression yields of A6 TCR variants in HEK293-6E system with his-tag appended on either α- or β-chain after transfection with different ratios of α- to β-chain.

Supplementary Figure 2. Results of duplicate test expressions of cysteine-substituted mutants of A6 TCR analysed with SDS-PAGE.

Supplementary Figure 3. MS analysis of A6 TCR and the stabilized variant ACAC2/BVBC1.

Supplementary Figure 4. Concentration dependency of melting profile of wild-type A6 TCR determined with DSC.

Supplementary Figure 5. Storage stability of wild-type and the stabilized variant ACAC2/BVBC1 of TCR A6 (left), DMF5 (center) and 1G4 (right) analyzed with SDS-PAGE after the incubation for the indicated number of days (M: Mark12 molecular weight marker).

Supplementary Figure 6. A: Storage stability of A6 TCR and the stabilized variant A6_ACAC2/BVBC1 analyzed with SDS-PAGE at 4, 20 and 37 °C (from left to right) after the incubation for the indicated number of days; B: storage stability of A6 TCR and mutants A6_ ACAC2 (left) and A6_BVBC1 (right) at 50 °C after the incubation for the indicated number of days (M: Mark12 molecular weight marker).

Supplementary Table 1. Amino acid sequence of A6DMF5 and 1G4 TCRs and their respective cognate peptide sequences used. The colors correspond to graphical representation of TCR mutants in Figure 1.

Supplementary Table 2. A6 TCR mutants proposed for mutagenesis with novel pairs of cysteine residues.

Supplementary Table 3. Supplementary Table 3. List of oligonucleotides used for construction of cysteine-substituted mutants of A6 TCR. For the N121C mutation, a different pair of oligonucleotides was used for 1G4 TCR.
